# Organic anion transporters, OAT1 and OAT3, are crucial biopterin transporters involved in bodily distribution of tetrahydrobiopterin and exclusion of its excess

**DOI:** 10.1007/s11010-017-3060-7

**Published:** 2017-05-22

**Authors:** Akiko Ohashi, Kaori Mamada, Tomonori Harada, Masako Naito, Tomihisa Takahashi, Shin Aizawa, Hiroyuki Hasegawa

**Affiliations:** 1 Department of Anatomy, Nihon University School of Dentistry, 1-8-13, Kanda-Surugadai, Chiyoda, Tokyo, 101-8310 Japan; 2 Division of Functional Morphology, Dental Research Center, Nihon University School of Dentistry, Chiyoda, Tokyo, 101-8310 Japan; 3 Department of Biosciences, Teikyo University of Science and Technology, Uenohara, Yamanashi 401-0193 Japan; 4 Division of Anatomical Science, Department of Functional Morphology, Nihon University School of Medicine, Itabashi, Tokyo, 173-8610 Japan

**Keywords:** Tetrahydrobiopterin, Dihydrobiopterin, Biopterin transport, NOS dysfunction, Organic anion transporter, Equilibrative nucleoside transporter

## Abstract

Tetrahydrobiopterin (BH_4_) is a common coenzyme of phenylalanine-, tyrosine-, and tryptophan hydroxylases, alkylglycerol monooxygenase, and NO synthases (NOS). Synthetic BH_4_ is used medicinally for BH_4_-responsive phenylketonuria and inherited BH_4_ deficiency. BH_4_ supplementation has also drawn attention as a therapy for various NOS-related cardio-vascular diseases, but its use has met with limited success in decreasing BH_2_, the oxidized form of BH_4_. An increase in the BH_2_/BH_4_ ratio leads to NOS dysfunction. Previous studies revealed that BH_4_ supplementation caused a rapid urinary loss of BH_4_ accompanied by an increase in the blood BH_2_/BH_4_ ratio and an involvement of probenecid-sensitive but unknown transporters was strongly suggested in these processes. Here we show that OAT1 and OAT3 enabled cells to take up BP (BH_4_ and/or BH_2_) in a probenecid-sensitive manner using rat kidney slices and transporter-expressing cell systems, LLC-PK1 cells and *Xenopus* oocytes. Both OAT1 and OAT3 preferred BH_2_ and sepiapterin as their substrate roughly 5- to 10-fold more than BH_4_. Administration of probenecid acutely reduced the urinary exclusion of endogenous BP accompanied by a rise in blood BP in vivo. These results indicated that OAT1 and OAT3 played crucial roles: (1) in determining baseline levels of blood BP by excluding endogenous BP through the urine, (2) in the rapid distribution to organs of exogenous BH_4_ and the exclusion to urine of a BH_4_ excess, particularly when BH_4_ was administered, and (3) in scavenging blood BH_2_ by cellular uptake as the gateway to the salvage pathway of BH_4_, which reduces BH_2_ back to BH_4_.

## Introduction

(6*R*)-L-*erythro*-Tetrahydrobiopterin (BH_4_) is an essential coenzyme of a group of aromatic amino acid hydroxylases [1–3]. BH_4_ is also required by nitric oxide synthases (NOSs) both for enzyme catalysis [4] and for functional dimerization [5]. Further, BH_4_ is the coenzyme of alkylglycerol monooxygenase which catalyzes irreversible ether lipid metabolism [6–8]. BH_4_ is autogenously synthesized in various cells which require this compound as the coenzyme. Inherited BH_4_ deficiencies are characterized by hyperphenylalaninemia and defective biosynthesis of classic monoamines such as dopamine, noradrenaline, adrenaline, as well as serotonin. BH_4_ supplementation ameliorates hyperphenylalaninemia in cases of inherited BH_4_ deficiency [9, 10] and also benefits patients with BH_4_-responsive phenylketonuria [11]. BH_4_ therapy using 6RBH_4_ has been successful in replacing BH_4_ except in the brain. Once 6RBH_4_ is administered to animals, presumably including humans, a certain portion is utilized in replacing innate BH_4_ and is integrated through endogenous metabolic pathways, similar to the intake of vitamins. The most common use of 6RBH_4_ to date is as an orphan drug for these inherited diseases [12–14]. However, like most drugs or supplements, its retention in the body is inefficient. According to the guidelines for BH_4_ therapy [15], the recommended dose is 5–15 mg/kg of 6RBH_4_ a day, roughly 600 mg for a 60 kg adult patient. These doses are over several hundred times greater than the 0.98 mg lost daily in urinary excretion [16] which is likely close to the amount of BH_4_ synthesized daily in healthy humans. BH_4_ replacement has also drawn increased attention with respect to whether it ameliorates NOS dysfunction in the cardio-vascular system. NOS dysfunction is largely caused by an increase in BH_2_ relative to BH_4_, a type of oxidative stress, rather than by a deficiency in BH_4_ [17]. One simple hypothesis was that supplying BH_4_ in an amount exceeding the endogenous BH_4_ level would pull the redox balance of BH_2_ and BH_4_ in favor of a relative BH_4_ increase. This hypothesis was likely based on the assumption that the administered BH_4_ would accumulate in the cell interior keeping its tetrahydro-form. This approach to ameliorating cardio-vascular symptoms caused by NOS dysfunction has had limited success to date [18]. In recent reports of trials on portal hypertension [19, 20], the authors concluded that “Sapropterin (6RBH_4_·2HCl) markedly increased tetrahydrobiopterin (BH_4_) levels, but also levels of its oxidized forms, which may counteract its potential beneficial effects.”

In general, an intracellularly functional and hydrophilic compound such as BH_4_ might be impermeable to the lipid bilayer of the cell membrane. Needless to say, permeation of BH_4_ across the cell membrane might require appropriate transporters. Although our knowledge of BH_4_ transport is incomplete, we attempted to clear some of this ambiguity by characterizing the relevant transporter(s) involved in the BH_4_ transport system. The work addresses three vital areas, A, B, and C, of which the core processes have been unclear due to a lack of knowledge of the crucial transporter(s) involved. **A**. Short retention of administered BH_4_. The extreme inefficiency seen after BH_4_ administration was likely brought about by the short retention of BH_4_ in the body caused by its massive exclusion into urine and feces; about a 90% gross urinary exclusion, which exceeded more than 60% of the dose, and took place within 2 h in rats [21, 22]. The rapid exclusion to urine essentially occurred via renal tubular secretion, namely, by trans-cellular transport across the tubular epithelial cell layer. These processes obviously involved a form of high-capacity transporter activity. The process was most probably mediated by at least two transporters acting in series, one engaging in uptake on the vascular side in a manner sensitive to probenecid (PBC) and the other controlling secretion to the urine on the lumenal side in a manner sensitive to cyclosporine A (CSA). **B**. Increase in BH_2_ after BH_4_ administration. The exogenous BH_4_ was delivered throughout the body after systemic oxidation to 7,8BH_2_, and the BH_2_ was then reduced back to BH_4_ by the salvage pathway, resulting in the accumulation of BH_4_ in target organs such as the liver and kidney [23–26]. Along this line of exploration, we discovered that ENT1 and ENT2, representatives of equilibrative nucleoside transporter families SLC29A1 and SLC29A2, respectively, were appropriate transporters for providing a gateway for sepiapterin (SP) and 7,8BH_2_ to enter the BH_4_ salvage pathway, an essential step in cell-to-cell inter-cellular BH_4_ redistribution [27, 28]. The ENTs were suspected of being inadequate for such high-capacity transport in the kidney after BH_4_ administration which represents a highly unnatural event. The putative transporters were distinct from the ENTs which are sensitive to nitrobenzylthioinosine (NBMPR) but less sensitive to PBC. In other words, other transporters must be furnished within the kidney to enable it to proceed with the massive uptake of biopterin species (BP), including BH_4_ and its oxidized form BH_2_, which are virtually all derived from the administered 6RBH_4_. Consequently, they were secreted by the kidney cells to the urine. **C**. Systemic BH_2_ scavenging mechanism. In our latest work on the pharmacokinetics of BH_4_ administration, PBC-sensitive transporter(s) was/were shown to be the key transporter in removing BH_2_ from the blood [29]. It was also suggested that the PBC-sensitive transporter played a key role in lowering the BH_2_/BH_4_ ratio which would otherwise be raised by BH_4_ administration.

We searched for a transporter which was able to uptake BH_4_ in a PBC-sensitive manner in the kidney. Here, we report that OAT1 and OAT3, representatives of the organic anion transporter families SLC22A6 and SLC22A8, are the plausible biopterin transporters engaging in massive BH_4_ exclusion in the kidney after BH_4_ administration. These transporters have been localized at the basolateral membrane of renal tubular epithelium, and they are both sensitive to PBC and have characteristic substrate specificities but with an overlapping preference [30–32]. Their fundamental features as transporters at the molecular level, such as their substrate selectivity, tissue distribution, and localization of their gene expression, have been extensively studied (for reviews [33–35]). Accordingly, we first examined whether renal epithelial cells were able to take up BH_4_ using kidney slices. We then examined whether rOAT1- and rOAT3-expressing LLC-PK1 cells of renal epithelial origin exhibited specific uptake of BH_4_, BH_2_ and SP. We confirmed their preference for BH_2_ and SP, precursors of the BH_4_ salvage pathway, using a *Xenopus* oocyte system expressing the respective transporters. Our finding that OAT1 and OAT3 were well suited to acting as a gateway of the salvage pathway of BH_4_ biosynthesis prompted us to conclude that these transporters played a crucial role in lowering the BH_2_/BH_4_ ratio by scavenging BH_2_ after BH_4_ administration. As one would expect, the OAT inhibitor PBC, when administered to healthy rats, raised blood BP levels not by BH_4_ supplementation but by a reduction in the baseline exclusion of endogenous BP in the urine.

## Materials and methods

(6*R*)-L-*erythro*-5,6,7,8-Tetrahydrobiopterin dihydrochloride (6RBH_4_·2HCl) was donated by Suntory (Asubio Pharma, Kobe, Japan) and sepiapterin (SP: 6-lactoyl-7,8-dihydropterin) and 7,8-dihydrobiopterin (BH_2_) were purchased from Schircks Laboratories (Jona, Switzerland). Methotrexate (MTX) was purchased from Wako (Osaka). Probenecid (PBC: 4-(dipropylsulfamoyl)benzoic acid), *p*-aminohippuric acid (PAH), penicillin G (PCG), cimetidine (CIM), and estrone sulfate (ES) were obtained from Sigma-Aldrich (St. Louis, MO). Working solutions of hydrophobic chemicals (100-fold concentration over the final concentration) were usually dissolved in an appropriate amount of DMSO and diluted in isotonic salt solution, and the pH of the medium was made neutral if needed. Collagenase (for *Xenopus* oocyte defolliculation) was purchased from Wako Pure Chemical Industries (Osaka, Japan).

### BH_4_ uptake experiments using rat kidney slices

Rats (SD: Sprague–Dawley) were obtained from Japan SLC (Hamamatsu, Japan). Kidney slices were prepared essentially according to Wedeen and Weiner [36] and BH_4_ uptake studies were carried out as described [31] in Prof. Sugiyama’s laboratory, Tokyo University, Department of Pharmaceutical Science. In brief, slices (0.3 mm thick) of whole kidneys from male rats were put in an ice-cold oxygenated incubation buffer containing 120 mM NaCl, 16.2 mM KCl, 1 mM CaCl_2_, 1.2 mM MgSO_4_, and 10 mM NaH_2_PO_4_/Na_2_HPO_4_ adjusted to pH 7.5. Two slices, together weighing 10–20 mg, were randomly selected and then incubated in a 12-well plate with 1 mL of oxygenated incubation buffer containing 6RBH_4_ in the presence of 1 mM dithiothreitol and other compounds. After incubation at 37 °C for 15 min, they were then rinsed with ice-cold buffer, blotted, and weighed. The slices were soaked in 100 µL of 0.1 M HCl and they were then frozen in liquid nitrogen. Biopterin contents were determined the next day after acid- or alkaline-I_2_ oxidation as described below.

### PBC administration to rats and collection of blood, urine, and liver and kidney tissues

The experimental procedure using rats was almost the same as described in the previous paper [29] except that the rats did not receive 6RBH_4_. In brief, rats (SD, 8–10-week-old males) were loaded with PBC (200 mg/kg) under anesthesia. Blood and urine were collected for BP determination from individual rats at designated times under long-lasting anesthesia on a warm gel pad for 6 h. The 0-time samples were taken from rats before the drug administration. At 6 h after PBC dosing, the rats were sacrificed to dissect the liver and kidney for BP determination.

### Uptake by cells under a monolayer culture in 96-well culture plates

LLC-PK1 cells were a generous gift of Dr. Naohiko Anzai, Kyorin University School of Medicine, and rOat1- and rOat3-transfected LLC-PK1 cells [37] were kindly donated by Dr. Hiroyuki Kusuhara (Graduate School of Pharmaceutical Sciences, University of Tokyo). The OAT-transfected cells were maintained as monolayer cultures in Dulbecco’s modified Eagle’s medium (DMEM, GIBCO^®^ Invitrogen) containing 5% fetal calf serum and G418 (400 µg/mL) at 37 °C in 5% CO_2_/95% air. The non-transfected cells were maintained similarly but without G418. Cells of this naïve cell line were used as the control of the transfected LLC-PK1 cells.

LLC-PK1 cells were plated on a 96-well analytical culture plate (Falcon 3072) and grown to a confluence of 4 × 10^4^ cells/well with 200 µL of the culture medium the day before the experiments. Prior to the uptake experiments, cells were adapted to a “basal culture medium” for 15 min. The “basal culture medium” was a modified Hank’s balanced salt solution which consisted of 137 mM NaCl, 5.37 mM KCl, 0.34 mM Na_2_HPO_4_, 0.44 mM KH_2_PO_4_, 0.34 mM K_2_HPO_4_, 5.5 mM glucose, and 5 mM HEPES, pH 7.4. Most transport experiments were conducted with reagents in the basal medium (100 µL) containing 1 mM dithiothreitol. Removal of the culture medium, leaving cells attached to the substrate, was performed by sucking off the medium with an 18-gauge needle (connected to an aspirator) inserted vertically and lightly touching the bottom of the culture plate [27]. Additional reagents such as pterin substrate (6RBH_4_, BH_2_, or SP) with or without other transporter ligands were introduced into the wells following a rinse and replacement with the new medium. Cellular uptake of particular components was allowed for designated times, and the uptake was terminated by a thorough change of medium without the substrate. Cells were then left in the substrate-free medium for 5 min, and subjected to three repeated rinses with ice-cold Ca^2+^- and Mg^2+^-containing phosphate-buffered saline (PBS(+)).

### Expression of hOAT1 and hOAT3 in *Xenopus* oocytes

African clawed frogs, *Xenopus laevis*, were purchased from Hamamatsu Seibutsu Kyozai (Hamamatsu, Japan). The gonads were dissected under ice anesthesia and subjected to collagenase treatment (1 mg/mL, 1 h). Mature oocytes were then subjected to manual defolliculation, essentially according to Bianchi and Driscoll [38].

Vectors hOAT1 and hOAT3 [39] were donated by Dr. A. Anzai, Kyorin University, School of Medicine. They were inserted into pcDNA3.1 (Invitrogen). Complementary RNAs (cRNAs) of hOAT1 and hOAT3 were prepared by in vitro transcription with T7 RNA polymerase in the presence of ribonuclease inhibitor and an RNA cap analog using an mMESSAGE mMACHINE kit (Ambion, Austin, TX). Defolliculated oocytes were injected with 50 ng of the respective cRNAs or the same volume of water as the control and incubated in modified Barth’s solution (82.5 mM NaCl, 2 mM KCl, 1 mM MgCl_2_, and 5 mM HEPES, pH 7.4) at 19 °C for 2 days.

### Transport experiment with *Xenopus* oocytes

Five oocytes each were placed in 100 µL of ND96 buffer (96 mM NaCl, 2 mM KCl, 1 mM MgCl_2_, and 5 mM HEPES, pH 7.4) containing 1 mM dithiothreitol in the wells of a 96-well plate at 25 °C for 60 min. Pterin uptake was initiated by replacing the medium with 100 μL of solution containing the desired concentration of the required ligands. The uptake was terminated at designated times by washing the oocytes three times with ice-cold ND96 buffer followed by the addition of 70 μL of acid-I_2_ or alkaline-I_2_ solution, as described below, for biopterin analysis. Subsequently, the oocytes were crushed evenly using a plastic rod with a flat tip (5-mm diameter), and allowed to oxidize for 60 min. They were then mixed with 70 μL of 4% ascorbic acid in 4 M HClO_4_ and cooled on ice for 1 h. Precipitates were removed by centrifugation. A slight turbidity remained and was removed by filtering the supernatant through a 3 mm cotton ball using a yellow-tipped pipette (Gilson) pressed against the bottom of the plastic tube (600-μL Eppendorf-type). The clear supernatant was then subjected to HPLC analysis. The endogenous biopterin, 0.020–0.025 pmol/oocyte, was disregarded.

### Determinations

Biopterin was determined essentially according to Fukushima and Nixon [40] as described previously [27]. Sepiapterin was not detectable in the extracts of LLC-PK1 or *Xenopus* oocytes under ordinary conditions. Even after SP was supplied, it was negligibly small in amount, presumably due to the high endogenous activity of sepiapterin reductase [28]. In this study, therefore, uptake of SP and BH_2_ by LLC-PK1 cells or *Xenopus* oocytes was determined indirectly using the amount of biopterin present after the acidic oxidation, i.e., the sum of BH_2_ and BH_4_.

The BH_4_ uptake by a given biological sample was expressed as the clearance, using the distribution volume (*V*_*d*_):

$${\text{BP uptake}} = V_{d} \div {\text{ time }} \div {\text{ tissue amount}},$$

where

$$V_{d} = {\text{BP}}_{{({\text{increase}})}} \left( {\text{pmol}} \right) \, \div \, \left[ {{\text{BH}}_{ 4} } \right]_{{({\text{out}})}} \left( {\upmu {\text{mol}}/{\text{L}}} \right) = {\text{BP}}_{{({\text{increase}})}} \div \, \left[ {{\text{BH}}_{ 4} } \right]_{{({\text{out}})}} \left( {\upmu {\text{L}}} \right).$$

In the case of BP uptake by the kidney slices at 15 min in the presence of extracellular BP, [BP]_(out)_ (µM), the BP uptake was expressed as:

$${\text{BP uptake}} = {\text{BP}}_{{({\text{increase}})}} /\left[ {\text{BP}} \right]_{{({\text{out}})}} \left( {\upmu {\text{L}}/\left( { 1 5 { \hbox{min} } \cdot {\text{mg tissue}}} \right)} \right).$$

For the BP uptake by LLC-PK1 cells in the presence of extracellular BP, [BP]_(out)_ (µM) for example, the BP uptake per hour per well of confluent cells was expressed as:

$${\text{BP uptake}} = {\text{BP}}_{{({\text{increase}})}} /\left[ {\text{BP}} \right]_{{({\text{out}})}} \left( {{\text{nL}}/\left( {{\text{h}} \cdot {{4\times 10^4 \text{cells}}}} \right)} \right).$$

For the *Xenopus* oocytes adapted to the experimental procedure (5 cells per assay):

$${\text{BP uptake}} = {\text{BP}}_{{({\text{increase}})}} /\left[ {\text{BP}} \right]_{{({\text{out}})}} \left( {{\text{nL}}/\left( {{\text{h}} \cdot {\text{oocyte}}} \right)} \right).$$

### Statistics

Statistical significance was analyzed by Student’s *t*-test or Williams’ test. The significance of difference between determinations at different times with individual animal groups was analyzed by a paired *t*-test. The significance of difference between three groups was analyzed by Holm’s test. All data were statistically analyzed using Pharmaco Basic Ver.15.0.1 (Scientist Co. Ltd., Tokyo).

## Results

### BH_4_ uptake by rat kidney slices

**Fig. 1:**
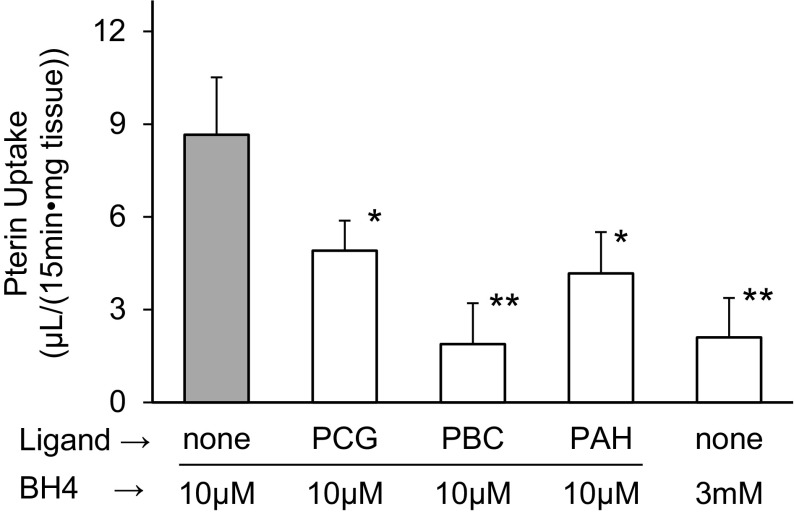
Uptake of 6RBH_4_ by kidney slices in the presence or absence of typical ligands of organic anion transporters. The kidney slices were prepared as described in “Materials and methods” section. The slices took up 6RBH_4_ and the uptake was inhibited by OAT ligands. The reagents used were 6RBH_4_ (10 µM and 3 mM), penicillin G (PCG, 1 mM), probenecid (PBC, 1 mM) and *p*-aminohippuric acid (PAH, 1 mM). The uptake of BH_4_ for 15 min was expressed as a portion of the clearance. *P < 0.05, **P < 0.01 (Holm’s test); each point represents the mean ± S.D. (n = 3–7)

The rat kidney slices taken from the cortex area contained 2.67 ± 0.78 nmol of BP per mg tissue weight, ca. 4-fold more than the whole kidney average (0.68 ± 0.11 nmol/g, *P* < 0.01). In the uptake experiment, the BP content of the slices increased almost linearly for more than 20 min, 79.5 ± 32.2 nmol/mg at 15 min in the presence of 10 µM 6RBH_4_, and the clearance was calculated to be 8.7 ± 3.2 µL/(15 min·mg) as depicted in Fig. 1. At a very high concentration of 6RBH_4_ (3 mM), the uptake was significantly decreased (*P* < 0.01), suggesting that the process was saturable with regard to BH_4_, consistent with the carrier-mediated process but not with physicochemical diffusion. Moreover, the process was inhibited by a group of organic anion transporter ligands [31, 32, 37]; namely, through strong inhibition by PBC (80%, *P* < 0.01) and moderate inhibition by PCG (40%, *P* = 0.040), a preferred substrate of OAT3, or PAH (50%, *P* = 0.024), a preferred substrate of OAT1.

Kidney slices are known to be one of the best functional systems in vitro with regard to their characteristic uptake which takes place exclusively at the basolateral membrane due to occlusion at the cut ends of the tubular cross section [36]. Accordingly, BH_4_ uptake by the slices in this manner was accounted for by the basolateral uptake of tubular epithelium. Furthermore, the 80% suppression by PBC strongly suggested that the relevant transporters involved in the BH_4_ uptake were mostly the organic anion transporters OAT1 and OAT3 which were reported to participate in the removal of various water-soluble compounds in plasma [30–32]. Although the involvement of other transporters in BH_4_ removal was not ruled out, the near exclusive participation of the PBC-sensitive transporters in the removal was suggested, and it might be a prerequisite for the rapid release of these compounds to the urine at proximal tubules in rats as observed previously [21, 22, 29].

### BH_4_ uptake by rOat1- or rOat3-transfected LLC-PK1 cells

**Fig. 2:**
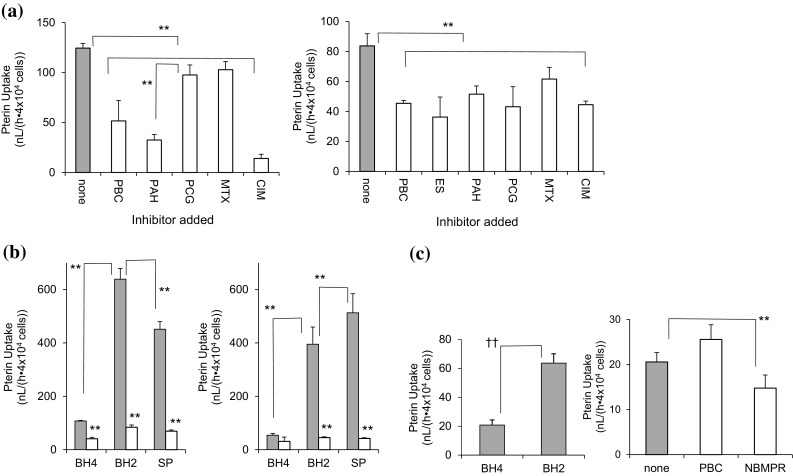
Biopterin uptake by rOat1- and rOat3-expressing LLC-PK1 cells and naïve LLC-PK1 cells. Tetrahydrobiopterin uptake by OAT-expressing or naïve LLC-PK1 cells was examined under a monolayer culture (4 × 10^4^ cells/well, 96-well analytical culture plate). **a** LLC-PK1 cells transfected with rOat1 (*left*) or with rOat3 (*right*) were used. The cells were exposed for 1 h to 50 µM 6RBH_4_ in the absence (*gray bars*, control labeled “none”) or in the presence (*open bars*) of OAT ligands (1 mM each except for methotrexate (MTX) at 80 µM). The rOat1- and rOat3-expressing LLC-PK1 cells took up 6RBH_4_ and the uptake was inhibited by the ligands of OAT1 and OAT3. The OAT ligands used were probenecid (PBC), estronesulfate (ES), *p*-aminohippuric acid (PAH), penicillin G (PCG), methotrexate (MTX) and cimetidine (CIM). **b** rOat1-LLC-PK1 cells (*left*) or rOat3- LLC-PK1 cells (*right*) were given 50 µM each of 6RBH_4_, dihydrobiopterin (BH_2_) or sepiapterin (SP) in the absence (*gray bars*) or presence (*open bars*) of 1 mM PBC for 1 h. The resultant biopterin accumulations of BH_2_ + BH_4_ were then compared between those in the absence of PBC *vs* the presence of PBC, and levels of BH_4_ *vs.* BH_2_ and of BH_2_ *vs* SP. **c** Uptakes of 6RBH_4_ and BH_2_ (50 µM each) by naïve LLC-PK1 cells were also compared (*left panel*). The uptake of 6RBH_4_ (50 µM) for 1 h (*right panel*) was analyzed in the absence (*gray bar*, labeled “none”) or presence (*open bars*) of 200 µM nitrobenzylthioinosine (NBMPR) or 1 mM PBC. The uptake of the pterins was expressed as a portion of the clearance. *P < 0.05, **P < 0.01 (Holm’s test); ^††^ *P* < 0.01 (Student’s *t*-test); each point represents the mean ± S.D. (n = 5–6)

Transport of BH_4_ by OAT1 and OAT3 and of BH_2_ and SP was characterized using rOat1- or rOat3-transfected LLC-PK1 cells, a cell line derived from the kidney of a male pig [41]. As shown in Fig. 2a, rOat1-LLC-PK1 and rOat3-LLC-PK1 cells took up 6RBH_4_, and these processes were inhibited by known ligands of OAT1 and OAT3. The BH_4_ uptake was strongly inhibited in rOat1-LLC-PK1 cells by the typical ligands of OAT1, PAH and cimetidine. On the other hand, BH_4_ uptake by the rOat3-LLC-PK1 cells was inhibited by the ligands of OAT3, ES and PCG. These results are consistent with the reported preference of the respective compounds for rOat1 and rOat3 [31, 32, 37]. The respective uptakes of 6RBH_4_, BH_2_ and SP by these cells were compared in the presence or absence of PBC (1 mM, Fig. 2b) to ensure that the PBC-sensitive portions of the pterin uptake were distinct from the other processes. Both rOat1-LLC-PK1 and rOat3-LLC-PK1 cells took up BH_2_ and SP, the precursors of the BH_4_ salvage pathway, in a PBC-sensitive manner and much more efficiently than their uptake of 6RBH_4_. The uptake of all three pterins in the presence of PBC was minor and it may have been mediated by transporters other than OAT1 or OAT3. Naïve LLC-PK1 cells were capable of taking up 6RBH_4_ to a lesser extent than the above transfected cells but they were insensitive to PBC. The naïve cells took up BH_2_ in preference to BH_4_ (Fig. 2c, left). The endogenous BH_4_ uptake by these cells was around 20.7 ± 3.60 nL/(h·4 × 10^4^ cells) and was significantly inhibited by nitrobenzylthioinosine (NBMPR), a typical ENT ligand (*P* < 0.01) but not by PBC (Fig. 2c, right) suggesting that the uptake was mainly mediated by ENT1, ENT2 as described previously [27, 28]. Hence, our observations of the pterin uptake in the presence or absence of PBC using OAT-transfected LLC-PK1 cells, as shown in Fig. 2b, enabled us to distinguish between the endogenous transporters and those expressed as a result of the transfection.

### Transport of BH_4_, BH_2_, and SP by hOAT1- or hOAT3-expressing *Xenopus* oocytes

**Fig. 3:**
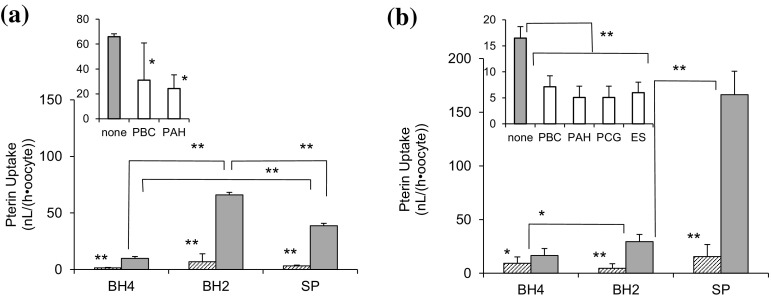
Uptake of 6RBH_4_, BH_2_, and sepiapterin by hOAT1- and hOAT3-expressing *Xenopus* oocytes. *Xenopus* oocytes were individually injected (*gray bars*) with 50 nL of hOAT1 (**a**) or hOAT3 (**b**) cRNA (1 ng/nL). As the control (*hatched bars*), oocytes were injected with 50 nL of distilled water. The oocytes were then allowed to express the respective transporters at 19 °C for 2 days. In the uptake experiment, all pterins were used at 50 µM. All oocytes injected with either cRNA took up significantly more pterins than the control and the uptake was inhibited by OAT ligands (1 mM each). The OAT ligands used were probenecid (PBC), *p*-aminohippuric acid (PAH), estronesulfate (ES) and penicillin G (PCG). **a** 6RBH_4_, BH_2_, and sepiapterin (SP) were taken up by hOAT1-expressing oocytes (*main panel*) for 1 h, and the BH_2_ uptake was analyzed in the absence (*gray bar*, labeled “none”) or presence (*open bars*) of OAT1 ligands (*upper panel*). (b) Uptake of 6RBH_4_, BH_2_, and SP by hOAT3-expressing oocytes (*main panel*) and inhibition of BH_4_ uptake by OAT3 ligands in the absence (*gray bar*, labeled “none”) or presence (*open bars*) of OAT3 ligands (*upper panel*). The uptake of the pterins was expressed as a portion of the clearance. *P < 0.05, **P < 0.01 (Holm’s test); each point represents the mean ± S.D. (n = 4–9)

*Xenopus* oocytes were separately injected with the cRNA of hOAT1 or hOAT3 and allowed to express the respective transporters at 19 °C for 2 days, as described in Materials and Methods. We observed an increased uptake of 6RBH_4_, BH_2_, and SP by all oocytes injected with the respective cRNAs, as shown in Fig. 3. The oocytes injected with hOAT1-cRNA showed the most pronounced uptake of BH_2_ followed by that of SP (Fig. 3a). Enhancement of 6RBH_4_ uptake was rather moderate when compared to that of BH_2_ or SP. The BH_2_ uptake was significantly inhibited by PBC (*P* < 0.01) and PAH (*P* < 0.01), both typical OAT1 substrates. In hOAT3-expressing oocytes, SP uptake was predominant followed by BH_2_ uptake (*P* < 0.01), while BH_4_ uptake was poorly enhanced, even when compared to BH_2_ (*P* < 0.01) (Fig. 3b). Nonetheless, the BH_4_ uptake was inhibited by OAT3 ligands, suggesting that it was also mediated by the hOAT3 expression product. Considering these results together with those obtained using rOAT-transfected LLC-PK1 cells, OAT1 of either rats or humans mediated the uptake of BH_2_ better than that of SP, and OAT3 mediated uptake of SP more than that of BH_2_. The pronounced preference for the dihydropterins 7,8BH_2_ and SP compared to BH_4_ seemed to be common to both OAT1 and OAT3 (cf. Figure 2b).

### Inhibition of OAT1 and OAT3 by probenecid and a decrease in bodily exclusion of endogenous BP in the rat

**Fig. 4:**
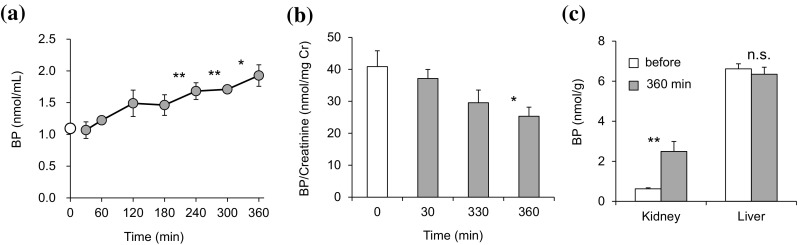
Elevation of blood BP accompanied by a decrease in the urinary loss of endogenous BP with a single dose of probenecid. Rats were given probenecid (PBC, 200 mg/kg, i.p.), and the blood (**a**) and urine (**b**) were collected sequentially from individual rats at the indicated times under sustained anesthesia for 6 h, then the kidney and liver (**c**) were dissected from the same rats at 6 h after the PBC dosing. The 0-time samples were taken from the rats without PBC treatment. The 0-time amounts of BH_2_ + BH_4_ (BP, *open symbols*) were compared with those of PBC-treated rat samples (*grey symbols*). In **a** and **b**, *P < 0.05, **P < 0.01 (“0-time” vs. “PBC-treated”, paired Student’s test), and in **c**, **P < 0.01, or n.s., no significant difference (“before” vs. “PBC-treated”, Williams’ test). Data are mean ± S.E. (n = 4)

In order to confirm that PBC prevents urinary exclusion of endogenous BH_4_, which was originally synthesized systemically de novo, by inhibiting the representative transporters, OAT1 and OAT3, we compared BP levels before and after PBC treatment in the circulating blood, in urine in the bladder, and in tissues of the kidney and liver. Rats were injected with probenecid (200 mg/kg, i.p.) under sustained anesthesia without BH_4_ administration. BP levels in the blood were gradually elevated to 1.6- and 1.8-fold within 6 h after PBC administration (Fig. 4a, *P* < 0.01). With the same rats, significant decrease was observed in the urinary BP contents, normalized with time-matched creatinine; and levels were about 30% less than those of the untreated rats (Fig. 4b, *P* < 0.05). Moreover, the tissue BP in the kidney of the same rats increased to 4.6-fold over the initial value (Fig. 4c, *P* < 0.01). Meanwhile, we did not observe any significant change in endogenous BP in the liver after the PBC treatment.

## Discussion

In earlier studies, we noted a facilitated clearance of BP by the kidney, particularly after 6RBH_4_ administration, and we demonstrated that it was due to the tubular secretion of exogenous BH_4_, distinct from removal by renal glomerular filtration [21, 22]. In our in vivo experiments analyzing the effect of PBC on the pharmacokinetics of administered 6RBH_4_ in rats, we showed that a PBC-sensitive transporter(s) enabled the liver and kidney to play their crucial role in bodily retention of BH_4_ and tubular secretion to the urine [29]. Organic anion transporters (OATs) are representative PBC-sensitive transporters known to participate in the uptake process in certain tissues such as the kidney and liver and to exhibit a particular localization. The role they play in kidney clearance of various xenobiotics and metabolic wastes has been well studied [30–32] (reviews [33–35]). We previously demonstrated that ENT1 and ENT2 were capable of transporting SP, BH_2_ and BH_4_, and that both were relevant as a gateway of the BH_4_ salvage pathway [27, 28]. ENTs comprise a family of equilibrative transporters that mediate the bidirectional permeation of nucleobases and similar heterocyclic compounds across biological membranes, indicating that BH_4_ and nucleotides share a common gateway for the salvage pathway. Their near ubiquitous distribution, including in endothelial cells, seemed to be appropriate for their body-wide role in biopterin distribution; however, more tissue-specific localization of vigorous transporters was expected for massive kidney-specific clearing. We therefore looked for other transporters which could play a major role in mediating biopterin permeation and clearance by the kidney. Accordingly, we focused our present search on tubular epithelium in examination of biopterin exclusion.

### OAT1 and OAT3 as biopterin transporters

Our first clue to uncovering the transporters responsible for biopterin uptake came from using kidney slices which took up BH_4_ and finding that this uptake was strongly inhibited by PBC together with other ligands of OAT1 and/or OAT3. It is also known that uptake by the basolateral side of tubular epithelium can be exclusively elicited in vitro using rat kidney slices [36]. Hence, we hypothesized that the transporters OAT1 and OAT3 were responsible for driving the renal exclusion of biopterin after systemic administration of 6RBH_4_. In order to prove this hypothesis, we employed an expression system using LLC-PK1 cells transfected with the rat OAT genes rOat1 and rOat3 following a method which established the functionality of these transporters in the uptake required for the exclusion of organic anions, nucleobases and nucleosides [31, 32]. As a result, the ability of these transporters was strongly suggested to mediate the cellular uptake of BH_4_, BH_2_, and SP. One uncertainty in this experimental system was that the naïve LLC-PK1 cells were able to take up BP to a noticeable extent. However, this fraction of BP uptake was thought to be mediated by other transporters such as the ENTs which are rather ubiquitous and essential for proliferation of cells in culture owing to their fundamental role as a gateway of the nucleotide salvage pathway. The ability of OAT1 and OAT3 to transport BH_4_, BH_2_ and SP was further confirmed by the uptake experiment using *Xenopus* oocytes expressing the human OAT genes hOAT1 and hOAT3. Although OAT1 and OAT3 both have a strong ability to transport BH_4_, BH_2_, and SP, this does not necessarily infer that they are the proprietary transporters in BP uptake. Instead, we consider that BP in plasma was targeted as a xenobiotic or metabolic waste to be eliminated by the kidney. The present result does not exclude the possible relevance to BP permeation of other transporters not examined here.

### Relevance of OAT1 and OAT3 in systemic BH_4_ metabolism

As for the body-wide relevance of OATs in BH_4_ metabolism, we observed a major movement of administered BH_4,_ including massive accumulation in the liver and rapid exclusion from the kidney [29]. Notably, in the liver and kidney, these processes were both inhibited by prior treatment with PBC. Based on these observations, we considered that the PBC-sensitive transporter(s) played a crucial role in enabling the liver to absorb and retain a large amount of BP and the kidney to take it up and exclude it in the urine. Renal trans-epithelial transport is composed of tandem permeations across the cell membrane, namely, uptake from the vascular side and release from the tubular lumen side, in which the former was strongly prevented by PBC and the latter, by CSA [21, 22]. In the present study, OAT1 and OAT3 were identified as the major PBC-sensitive transporters for BP exclusion. However, if they act too vigorously to exclude BP, this would raise concern that BP could be thoroughly removed from the body. In addressing this issue, we previously examined BP levels in urine and plasma separately from red blood cells. We noted that most of the plasma BP, whose level rose sharply after 6RBH_4_ administration, was rapidly and massively excluded in the urine by trans-cellular BP secretion which far exceeded glomerular filtration but only until the point at which the plasma BP had decreased to about 1 nmol/mL, 10-fold the endogenous level, and the level remained higher than the endogenous level for a period of hours [21, 22]. Considering the exclusion dynamics of exogenous BP, these transporters likely play the core role in removing extraordinarily high concentrations of plasma BP as a sort of protective mechanism. Although the transporter ligands CSA and PBC strongly blocked the exclusion of BP across the epithelium to the urine, they raised the blood BP to a very high concentration while they had no effect on the glomerular filtration of BP. The ligand treatment, therefore, did not improve the efficiency of BP replacement because the inhibition of the trans-cellular BP passage was offset by a more rapid outflow through glomerular filtration due to a compensatory elevation of plasma BP [29]. Despite the “low efficiency” of peripherally administered 6RBH_4_, this supplement can provide enough BH_4_ cofactor for pterin-dependent hydroxylases in peripheral organs, such as the liver, in patients with an endogenous BH_4_ deficiency [42–44]. 6RBH_4_ administration immediately elevates plasma BP levels. We consider that an extreme concentration higher than threshold was targeted for biological detoxification by means of the tubular secretion in which OAT1 and OAT3 played the essential role. Hence, the “low efficiency” in BH_4_ supplementation was a consequence of the blood BP elevation over the critical level, which was 10-fold the endogenous level in the case of rats, in the early period after 6RBH_4_ administration.

### Remarks on the relevance of OAT1 and OAT3 for systemic BH_2_ scavenging after BH_4_ supplementation

Contrary to general expectations, the administration of 6RBH_4_ initially caused an increase in BH_2_ in the circulation. However, the elevated BH_2_ ratio declined within a short time and dropped even further to a level lower than the initial level. An initial surge in BH_2_ and a sharp elevation of the BH_2_/(BH_2_ + BH_4_) ratio was first observed in the blood in a BH_4_ replacement experiment in which mice were intraperitoneally administered 6RBH_4_, 7,8BH_2_ or SP [23]. A rather long-lasting BH_2_ increase was also observed in rat plasma after intravenous administration of 6RBH_4_, subsequently, the elevated BH_2_ ratio in the plasma gradually returned to normal over a period of hours [29]. Presumably, the increased plasma BH_2_ had been removed by its selective uptake and was subsequently converted to BH_4_. This consequence was paradoxically illustrated by the finding that treating the rat with PBC prior to 6RBH_4_ administration strongly stimulated the BH_2_ increase in the blood and urine while it attenuated the BP increase in the liver and simultaneously retarded urinary BP excretion.

We have demonstrated in this work that both OAT1 and OAT3 drove the cellular uptake of the dihydro-forms of pterins, BH_2_ and SP, in roughly a 5- to 10-fold preference over the tetrahydro-form, BH_4_. The liver and kidney are undoubtedly well furnished with a BH_4_ salvage pathway as shown by the fact that the tissue BH_2_ ratio, BH_2_/(BH_2_ + BH_4_), in these organs did not significantly change even after the massive uptake of BH_2_ from the plasma after 6RBH_4_ administration [29]. BH_2_ uptake is a prerequisite of BH_2_ conversion to BH_4_ in the liver and of BH_2_ removal to the urine by the kidney. Furthermore, the strong preference of OAT1 and OAT3 for BH_2_ over BH_4_ made it much more efficient for these organs to perform their respective functions. Based on these reports together with the present observation regarding the transporter preference for BH_2_, we consider that OAT1 and OAT3 work as part of a body-wide machinery for scavenging BH_2_ and maintaining the redox balance, at least after BH_4_ administration. With regard to phenylalanine-, tyrosine-, and tryptophan hydroxylases, the relative increase in BH_2_ does not interfere with the enzyme activity to any significant extent, however, it does lead to a critical failure in NOS function.

Administration of 6RBH_4_ has been attempted as a translational medicinal approach to improving eNOS dysfunction, especially in cardio-vascular disorders. The dysfunction of eNOS is characterized by uncontrolled uncoupling of O_2_ reduction producing “reactive oxygen/nitrogen species” (ROS/RNS), and it is believed to be a great risk factor for cardio-vascular disease. Various encouraging experimental studies have been reported, however, these attempts have had limited success in trials to ameliorate human cardio-vascular dysfunction [18]. The binding of BH_2_ to active NOS causes an uncoupling of the O_2_ reduction involved in the enzyme reaction [45]. To avoid sustained eNOS dysfunction, the enzyme should not be exposed to a high concentration of BH_2_ relative to BH_4_ because the affinity of NOS for BH_2_ was reported to be not particularly low; the IC_50_ of BH_2_ to eNOS was comparable to that of BH_4_ [17] and the K_i_ of nNOS to BH_2_ was only 10-fold higher than to BH_4_ [46]. Hence, the success of 6RBH_4_ administration for improving NOS dysfunction might primarily depend on its lowering of the BH_2_/(BH_2_ + BH_4_) ratio rather than on raising the BH_4_ concentration. This has been noted by many researchers. As two examples, (1) the importance of the BH_4_ salvage pathway driven by dihydrofolate reductase has been well documented and the scavenging of BH_2_ was shown to be enabled by vigorous reductase activity [47–49]. The authors argued that endothelial cells were poorly furnished with dihydrofolate reductase, which made NOS function in these cells extremely vulnerable to BH_2_ produced in situ in the cell interior. It was also reported that (2) endothelial cells of human origin showed dramatically less dihydrofolate reductase activity compared to cells of other species including cows and mice [50]. Taken together, endothelial cells did not appear to have the means to scavenge BH_2_ in the local cell interior. Nonetheless, blood BH_2_ can be produced either near or at some distance away from local endothelial cells. In addition, endothelial cells readily take up plasma BH_2_ because of their asymmetrically dense expression of ENT2 on the apical membrane facing the vascular lumen side [51]. As an extreme example, after BH_4_ administration, eNOS of endothelial cells is vigorously exposed to a high BH_2_ concentration from the circulating blood. In this context, attenuation of systemic BH_2_ production or stimulation of BH_2_ scavenging has a direct impact on the eNOS dysfunction of endothelial cells.

This work has demonstrated that OAT1 and OAT3 are both involved in systemic BH_2_ scavenging in terms of their characteristic mediation of BH_2_ uptake and their known distribution on the vascular side of renal tubular endothelium. Effective BH_2_ scavenging in the kidney is obviously enabled by the high-capacity uptake of blood BH_2_ mediated by OAT1 and OAT3. Although OAT3 is the dominant OAT expressed in the liver [30], other OAT counterparts in this organ, such as OAT2 [33, 35], are also interesting but their roles remain elusive.

### Relevance of PBC-sensitive transporters in exclusion of endogenous BP in the urine

As a matter of general physiology, we are also interested in the relevance of OAT1 and OAT3 in the homeostasis of BH_4_ metabolism under ordinary conditions without exogenous BH_4_ supplementation. Our experiments addressed this issue (Fig. 4) and demonstrated that the administration of PBC to healthy rats significantly decreased BP exclusion in the urine which was accompanied by a rise of BP in the blood, with both levels measured using the same individual rats. Since there are no known reasons for the drug to stimulate de novo BP biosynthesis, the increase in the blood BP was accounted by a kickback from inhibition of the renal secretion of BP by PBC. These results suggested that the loss of BP through the urine strongly influences the endogenous amount of BP in the blood. We also observed that the BP content in the kidney was significantly increased in the presence of PBC. An almost proportional increase in kidney BP relative to blood BP was even more pronounced in our previous study in which rats were administered “6RBH_4_ alone” or “6RBH_4_ + PBC” [29]. The mechanism of this in vivo increase involving local OAT1 and/orOAT3 remains unclear. Since OAT1 and OAT3 are the only known PBC-sensitive transporters which enable BP to permeate across the cell membrane, it is highly plausible that OAT1 and OAT3, if not acting locally in the kidney, are involved in the mechanism controlling the bodily retention of BH_4_. However, this subject awaits further in vivo exploration.

As mentioned at the beginning of the Discussion, ENT1 and ENT2 have been identified as BP transporters. ENTs and OATs are similar in their preference for SP and BH_2_ as substrates rather than BH_4_. However, in contrast to the localization of OATs in specific tissues such as in the kidney and liver, ENTs are characterized by their near ubiquitous distribution including in endothelial cells. Generally speaking, it is likely that ENT1 and ENT2 assume their share of the mobilization of BP, including its precursor SP, in the body interior, while OAT1 and OAT3 deal with detoxication in response to a BP excess and act in removal of this pterin from the body.
